# *In Vitro* Cytotoxicity and *In Vivo* Acute and Chronic Toxicity of Xanthii Fructus and Its Processed Product

**DOI:** 10.1155/2013/403491

**Published:** 2013-11-26

**Authors:** Jie Yu, Mei-Zhen Song, Jing Wang, Yun-Fei Li, Pei Lin, Lin Que, Zhaorigetu Bao

**Affiliations:** Yunnan University of Traditional Chinese Medicine, Kunming, Yunnan 650500, China

## Abstract

Xanthii Fructus (XF), the fruit of *Xanthium sibiricum* Patr., was used in the treatment of rhinitis and related nasal disease. Adverse effects of Xanthii Fructus are frequently reported these years. In the paper, *in vitro* renal cytotoxicity and *in vivo* acute and chronic toxicity researches of Xanthii Fructus (XF) and its processed product (processed Xanthii Fructus (PXF)) were carried out. Water extraction of XF displayed no cell membrane damage effects even in the highest concentration (100 **μ**g/mL); however, it might affect the function of renal cell mitochondria. Acute toxicities were observed only in high and middle dosage groups. Fortunately, the single dose administration of XF or PXF was safe even at the highest daily dosage. Twelve-week chronic toxicity assays were performed in SD rats with low, middle, and high dosage. Notable changes in body weight and blood cell and BUN and Scr changes sporadically occurred in middle and high groups after the 9th week. Serum HA and HPCIII values were sustained increasing from the 4th week to the 8th week in Group V male rats, which indicated that the renal fibrosis risks still existed although no fibrosis was found in the pathological examination of the liver and kidney.

## 1. Introduction

Concerns about the toxicity of traditional Chinese medicines are increasing with each passing day after the “aristolochic acid nephropathy” case. Aristolochic acids are hypothesized to be causative agents in Balkan endemic nephropathy and a related possibly identical condition known as “Chinese herbs nephropathy” [1].

Hereafter, adverse effects of traditional Chinese medicine other than aristolochic acid related crude drugs, such as Xanthii Fructus, Polygoni Multiflori, and Leonuri Herba, are frequently reported [2–8]. Among them, Xanthii Fructus attracts our intensive attention due to its wide range of applications in the treatment of rhinitis, snuffle, nasosinusitis, and related nasal disease. Xanthii Fructus is the fruit of *Xanthium sibiricum* Patr. [9]. Xanthii Fructus is the most frequently used crude drug in compound recipes, which were used in the treatment of rhinitis. Xanthii Fructus is recorded as nontoxic in the ancient herbal “Shen Nong Ben Cao Jing” (Shen Nong's Classic of Materia Medica, composed in about AD 1082). Nevertheless, its toxicity is recognized gradually as time goes by. It is recorded as slight noxious or middle level noxious in most of the ancient herbals, such as Zheng Lei Ben Cao (composed in about AD 1082) and Pin Hui Jing Yao (composed in about AD 1505).

Unfortunately, although the toxicity of Xanthii Fructus is clearly alerted in the Pharmacopoeia of China (2010 edition) [9], poisoning even death cases are reported continuously after immoderate usage of Xanthii Fructus as food or medicine. The authors have reviewed the clinic toxicity reports of Xanthii Fructus systematically [7]. Three death cases caused by multiple organ failure were reported [3] in Turkey (no clear dosage reported). An outbreak characterized by vomiting and rapid progression to unconsciousness and death was reported in northeastern Bangladesh after the consumption of *Xanthium strumarium* seedlings in large quantities, due to inaccessibility of other foods [5]. Kidney damage was the most frequently reported adverse effect caused by Xanthii Fructus [7]. Hematuria, albuminuria, abnormal renal function, and acute renal failure were the most presented renal damage types [2, 4]. Judging from all the adverse effects reports, we find that most of these severe adverse effects are occurred in the high dosage or even overdose administration cases.

The toxicity of Xanthii Fructus is usually considered to be associated with two diterpenoids glycosides, atractyloside (AL) and carboxyatractyloside (CAL). The biochemistry and toxicology of them are well investigated and elucidated both in *in vitro* and *in vivo* [10, 11] methods. The LD_50_ of AL is 15 mg/kg in dog (*iv*), 434 mg/kg in mouse (*ip*), and 143 mg/kg in rat (*ip*). According to the latest literature [10], content of CAL decreased dramatically after processing procedure, while AL content increased after processing procedure. CAL was reported to be about 1.16 mg–2.25 mg/g in XF and 0–0.21 mg/g in PXF. On the other hand, AL could not be detected in XF; however, the content of AL increased to about 0.17–2.56 mg/g in PXF. However, the toxicity mechanisms involved are not well investigated.

Despite the prevalent reports of their toxicity, the toxicity research data about XF and PXF still need to be clarified, especially, their toxicity in normal or middle dosage administration case. In this paper, we systemically evaluated the *in vitro* and *in vivo* toxicity of Xanthii Fructus, its processed product, and its major chemical constituent.

*In vitro* renal cytotoxicities were investigated by human kidney HK-2 cell line, an immortalized proximal tubular epithelial cell line from normal adult human male kidney, retaining the phenotype and functional characteristics indicative of well-differentiated proximal tubular epithelial cell [12]. Not only the water extracts of Xanthii Fructus but also its major chemical constituents, atractyloside potassium salt (AL), were involved. Culture of this cell line has previously been employed by several studies to investigate nephrotoxicity of various toxic compounds [13–15].

Acute and chronic toxicities were tested in Kunming mice and SD rats, respectively. Single dosage of XF or PXF was given to Kunming mice in order to investigate their acute toxicity. Twelve-week chronic toxicity assay was also conducted in rats. Systemic toxicity indexes were involved in both acute and chronic toxicity assays in order to elucidate their toxicity comprehensively. Some kidney toxicity indexes and early indicators of renal fibrosis were involved in chronic toxicity assays.

## 2. Materials and Methods

### 2.1. Chemicals

3-(4,5)-Dimethylthiahiazo(-z-y1)-3,5-di-phenytetrazoliumromide (MTT) and trypsin (1 : 250) were acquired from Amresco, USA. Dimethylsulfoxide (DMSO) was purchased from Sigma, USA. Other reagents were all above analytical grade. Lactate dehydrogenase (LDH) assay kits were purchased from Changchun Huili Biotech Co., Ltd., China. BUN and Scr assay kits were purchased from Biosino Bio-technology & Science Inc., China. Enzyme-linked immunosorbent assay kits of HA, LN, and HPC III, three early indicators of renal fibrosis, were obtained from Cusabio Biotech Co. Ltd, Wuhan, China.

### 2.2. Processing Procedure of Xanthii Fructus

**Figure 1:**
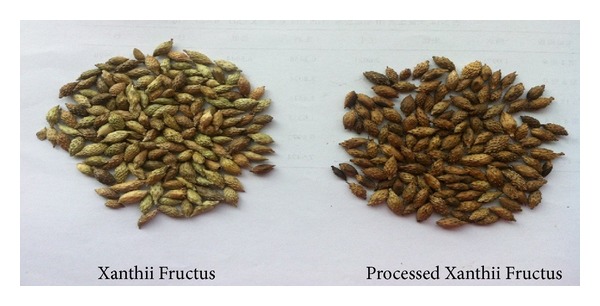
Photographs of Xanthii Fructus and its processed product.

Xanthii Fructus was purchased from Juhuacun Chinese herbal medicine market, Kunming, Yunnan, China, in February, 2009 (shown in Figure 1), and was identified as fruit of *Xanthium sibiricum* Patar. by the author, Associate Professor Jie Yu. Voucher specimens were deposited in the Herbarium of Pharmacognosy, Yunnan University of Traditional Chinese Medicine.

Processed Xanthii Fructus was stir-fried from Xanthii Fructus with mild fire to yellowish brown (Figure 1). Processing of Xanthii Fructus was carried out by the authors according to the procedures documented in the Pharmacopoeia of China, 2010 edition [9].

### 2.3. *In Vitro* Renal Cytotoxicity Assay

#### 2.3.1. Preparation of Exposure Solution of Atractyloside Potassium Salt for* In Vitro* Assay

**Figure 2:**
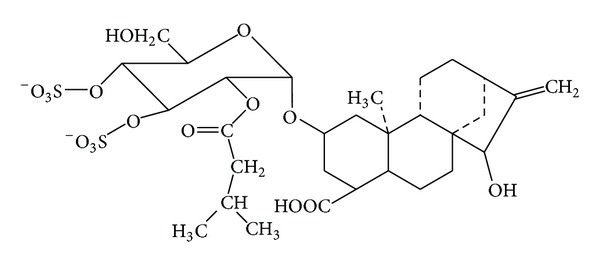
Structure of atractyloside potassium salt (AL).

Atractyloside potassium salt (AL), major constituent of XF and PXF, was purchased from Sigma, USA (structure shown in Figure 2), with purity higher than 98%. The exposure solutions of AL in concentration from 2.5 to 320 *μ*M were diluted by RPMI 1640 containing 0.2% fetal bovine serum (FBS).

#### 2.3.2. Preparation of Extraction of Xanthii Fructus for *In Vitro* Assay

40 g powder of XF was decocted with water (1000 mL, 800 mL, and 600 mL) for 3 times, respectively. The extractions were combined, condensed, and lyophilized. Extraction yield was 23.15%.

XF stock solution was achieved by dissolving them in DMSO at a concentration of 10 mg/mL. Then dissolving in DMSO, it was further diluted with 0.2% FBS-RPMI 1640 to graded concentrations from 10 to 100 *μ*g/mL.

#### 2.3.3. Culture Conditions

The whole research was carried out by HK-2 cells, immortalized proximal tubular epithelial cell line, which were purchased from Cell Bank, Kunming Institute of Zoology, Chinese Academic of Science. Cells were cultured in monolayer culture in DMEM medium (Gibco Invitrogen Corp.), supplemented with 10% fetal bovine serum (HyClone).

Cells were seeded at an appropriate cell density in a humidified incubator (Series II water jacketed CO_2_ Incubator Model 3111, Thermo Electron Corporation, USA) with 5% carbon dioxide and dioxide, 95% air at 37°C for different assay and left to grow to 80-90% confluence.

#### 2.3.4. MTT Cell Viability Assay

This assay procedure was adopted from the previous report [8]. HK-2 cells were seeded in 96-well plates (Corning Incorporated) at a cell density of 2 × 10^4^ in 200 *μ*L/well and then incubated for 48 h till 80–90% confluence. Then, the cell synchronization was routinely accomplished by incubating cells in G_0_ medium for 12 h before the test. Subsequently, cells were exposed to fresh G_0_ medium with various concentrations of test compounds or extractions for 24 h.

At the end of the incubation period, 10 *μ*L/well of 5 mg/mL MTT (Amresco, USA), which was dissolved in PBS (pH 7.4) and sterilized by filtrating through a Millipore filter (0.22 *μ*m), was added in medium for a further 4 h incubation; then the medium was removed and 100 *μ*L DMSO was added to dissolve the resulting intracellular purple formazan product. The 96-well plates were gently swirled for 5 min in room temperature. The absorbance was measured at 570 nm. This was conducted by a microtiter plate reader (DNM-9602G, Beijing Perlong Medical Equipment Co., Ltd.). The wells added with cell and culture medium but without stimulus were considered as normal control, and the wells added culture medium only were considered as blank control. HgCl_2_ (10 *μ*M), a typical renal toxicant, was used as a positive control. Each concentration of tested compounds or extractions was paralleled for three times and 6 wells were averaged in each plate. The survival rate of cells in each concentration was calculated.

#### 2.3.5. LDH (Lactate Dehydrogenase) Leakage Assay

This assay procedure was adopted from the previous report [8]. HK-2 cells were seeded in 24-well plates (Corning Incorporated) at a cell density of 1 × 10^4^ in 500 *μ*L/well and then incubated till the cells reached 80–90% confluence. Then, the cell synchronization was routinely accomplished by incubating cells in G_0_ medium for 12 h before the test. Subsequently, cells were exposed to fresh G_0_ medium with test compounds or extraction for 24 h. At the endpoint of the incubation period, all the culture medium was collected and the intracellular LDH was ingathered after lysing cells with 500 *μ*L 0.1% Triton X-100 (Sigma). The enzyme content in culture medium and cell lysis solution was analyzed by lactate dehydrogenase kit by an UV detector (725, Shanghai Spectrum Instruments Co., Ltd.). The leakage percentage was calculated by the extracellular LDH activity compared with the total LDH activity (extracellular + intracellular LDH). HgCl_2_ (10 *μ*M) was used as a positive control. Two wells were repeated in each 24-well plate and three plates were paralleled for different concentrations of each compound or extraction.

### 2.4. *In Vivo* Acute and Chronic Toxicity Assays

#### 2.4.1. Animals

Acute toxicity research was carried out on Kunming mice of both sexes. 70 Kunming mice were provided by the Experimental Animal Center of Yunnan University of Traditional Chinese Medicine. They were aged 5 weeks and weighed 18~22 g. Mice of the same sex were housed ten in a stainless steel cage containing sterile paddy husk as bedding in ventilated animal rooms.

Chronic toxicity research was carried out on SD rats of both sexes, which were also provided by the Experimental Animal Center of Yunnan University of Traditional Chinese Medicine. They were aged 6 weeks and weighed 200 ± 20 g. Rats of the same sex were housed four in a stainless steel rat cage containing sterile paddy husk as bedding in ventilated animal rooms.

All mice and rats were acclimated in the controlled environment (temperature 22 ± 1°C, 60 ± 10% humidity, and a 12 h/12 h light/dark cycle) with free access to water and a commercial laboratory complete food. All animal experiments were performed in compliance with the animal experimental ethics committee of Yunnan University of Traditional Chinese Medicine. All reasonable efforts were made to minimize the animals' suffering.

#### 2.4.2. Preparation of Extraction of Xanthii Fructus for* In Vivo* Toxicity Assay

500 g Xanthii Fructus was immersed in water for an hour. Then, they were decocted with water (2500 mL, 1500 mL, and 1500 mL) for 3 times, 30 mins per time, respectively. The extraction was combined and condensed to 10 g/mL (the maximum concentration that can flow through the gavage needle for mice). The same procedure was adopted in the preparation of processed Xanthii Fructus extraction.

The recommended dosages of XF and PXF were both 3–10 g/per day according to the Pharmacopoeia of the People's Republic of China, 2010 edition [9]. The low, middle, and high dosages of XF and PXF in acute toxicity assay for mice were 16.68 g/mL (10 times the highest human dosage), 166.8 g/mL (100 times the highest human dosage), and 400 g/mL (240 times the highest human dosage).

Much lower dosages were adopted in the chronic toxicity assay in order to imitate the human clinic medication practice of XF and PXF. In the chronic toxicity assay, the low, middle, and high dosages of XF and PXF were 0.585 g/mL (the same as the human median dosage), 1.755 g/mL (3 times the human dosage median dosage), and 5.265 g/mL (9 times the human dosage median dosage).

#### 2.4.3. Acute Toxicity Testing

All mice of both sexes were randomly divided into 7 groups of ten each (Table 1). The male and female rats were all five in each group. All mice fasted for 12 h before the administration of each agent. Group A received physiological saline only and served as vehicle. Group B to G received water of XF or PXF in low, middle, and high dosage, respectively (Table 1).

Clinical symptoms of mice after respective treatments were monitored closely for 4 hr after gavage. Seven-day observation was carried out on all the surviving mice. Mortalities, clinical signs, time of onset, duration, and reversibility of toxicity in each group were recorded. Gross necropsies were performed on all animals, including those sacrificed moribund, found dead, or terminated at 7 days. Routine analysis of blood cells in Groups A, B, C, E, and F was carried out after the sacrifice in the end of the research. For light microscopic observations, samples from liver, kidney, lung, and heart of each group were fixed in formalin fixative and processed routinely for embedding in paraffin. Both male and female mice samples were used in the histopathological examination. Tissue sections in 5 *μ*m thickness were stained with hematoxylin and eosin (H&E) and examined under a light microscope.

#### 2.4.4. Chronic Toxicity Assays

A 12-week chronic toxicity assay was executed to investigate the long term toxicity after normal dosage administration of XF and PXF every day. 56 rats of both sexes were randomly divided into 7 groups of eight each (Table 2). The male and female rats were all four in each group. Group I received physiological saline only and served as vehicle. Group II to IV received water extraction of XF, while Group V to VII got PXF water extraction in various dosages. All rats fasted for 2 h before administration of each agent every day.

Body weights were recorded every three days till the end. Samples of blood 1.5–2 mL in volume were collected from the retro-orbital venous plexus once every week throughout the study. Blood samples were collected under ether anesthetic condition, two hours after the administration of therapeutic agents in the morning. Routine analysis of blood cells was performed by hematology analyzer (URIT-2900, Guiling, China). Levels of BUN and Scr were determined by enzymatic colorimetric method using commercial assay kits (Biosino Bio-technology & Science Inc.) by AB-1020 automatic biochemical analyzer (Sunostik Medical Technology Co., Ltd.). As early indicators of renal fibrosis, serum HA, LN, and HPCIII content values were also tested in our research. They were analyzed using enzyme-linked immunosorbent assay kits from Cusabio Biotech Co., Ltd. (Wuhan, China). HA, LN, and HPCIII were tested only on weeks 4, 8, and 12. All bioassays were carried out in duplicate.

For light microscopic observations, samples from liver and kidney of each group were fixed in formalin fixative and processed routinely for embedding in paraffin. Both male and female samples were used in the histopathological examination. Tissue sections in 5 *μ*m thickness were stained with hematoxylin and eosin (H&E) and examined under a light microscope.

### 2.5. Statistical Analysis

All data in this research were expressed in the form of mean ± SD. One-way analysis of variance (ANOVA) was performed when multiple group comparisons were carried out. Results were classified into three significance levels using the *P* value of 0.05, 0.01, and 0.001.

## 3. Results

### 3.1. *In Vitro *Renal Cytotoxicity of Atractyloside Potassium Salt

In order to avoid overestimation or underestimation of the toxicity of a substance, incubations with various concentrations are required to distinguish between effects on specific organelles or general cytotoxicity. More than one assay should be used to determine cell viability in *in vitro* studies, as this would increase the reliability of the results obtained [8, 16]. In this research, both MTT and LDH assays, reflected the function of mitochondria and cell membrane, were adopted.

As listed in Table 3, AL showed no inhibition on the cell proliferation of HK-2 cells in MTT survival rate assay. The cell survival rate was not affected by the exposure of AL, and it almost remained at 100% even at the highest concentration of 320 *μ*M. Meantime, the LDH leakage rate was not affected by the exposure of AL, and it almost remained at 10% (similar to the control group) even at the highest concentration of 320 *μ*M.

Judging from these data, AL not only showed no inhibition on the cell proferliation of HK-2 cells but also exhibited no damage on the cell membrane function.

### 3.2. *In Vitro *Renal Cytotoxicity of Xanthii Fructus

**Figure 3:**
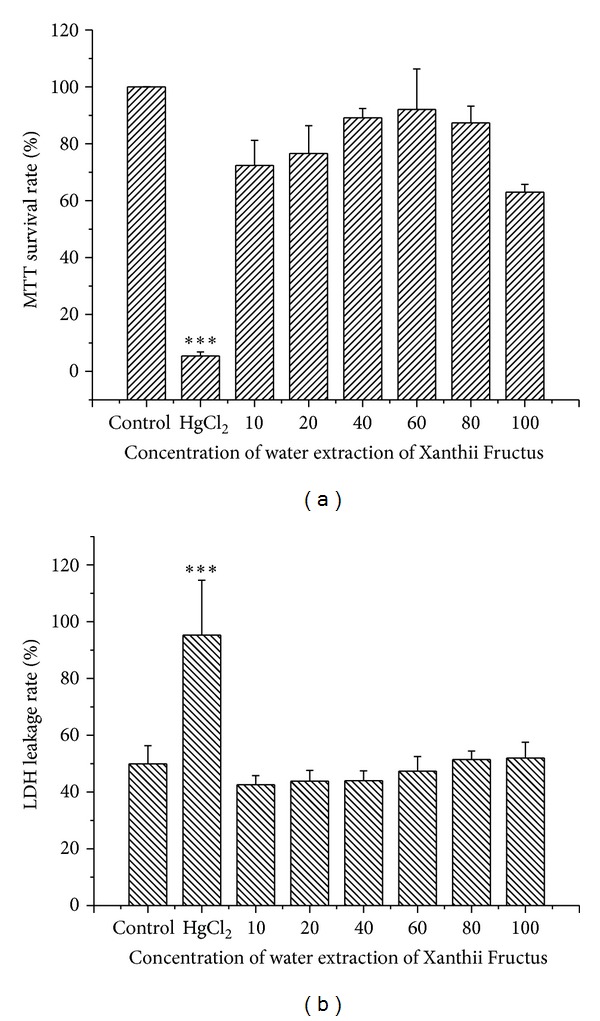
MTT survival rate (a) and LDH leakage rate (b) of HK-2 cell exposured to water extraction of Xanthii Fructus. The ∗ indicates a significant difference compared with control group, *P < 0.05, **P < 0.01, ***P < 0.001.

Results of renal cytotoxicity assay of water extraction of XF were shown in Figure 3. MTT survival rates ranged from 70% to 90% in the 10 to 80 *μ*g/mL XF water extraction. However, the survival rate decreased to 63.0 ± 2.7% in the 100 *μ*g/mL XF water extraction. Water extraction of Xanthii Fructus showed moderate inhibition effects on proliferation of HK-2 cells in high dosage (Figure 3(a)). On the other hand, we find that the HK-2 cell membrane remains undamaged even in the highest 100 *μ*g/mL XF water extraction exposure (Figure 3(b)).

### 3.3. *In Vivo *Acute Toxicity of XF and PXF

In the *in vivo* acute toxicity assay, close observations of mice clinic signs were made before dosing and up to four hours immediately after dosing. All animals were observed daily for convulsions, excitement, posture, piloerection, breathing difficulty, sedation, anorexia, diarrhea, hemorrhage, and death during the 7-day assay period.

Deaths were observed only in high dosage XF and PXF group. Mortality rate in XF group (80%) was significantly higher than that in PXF group (40%) in both sexes. No death was found in middle or low dosage group no death was found in middle or low dosage group. Regular acute toxicity symptoms and signs, such as salivation, slow action, and convulsions, were observed in middle or high XF and PXF groups (Table 4).

**Figure 4:**
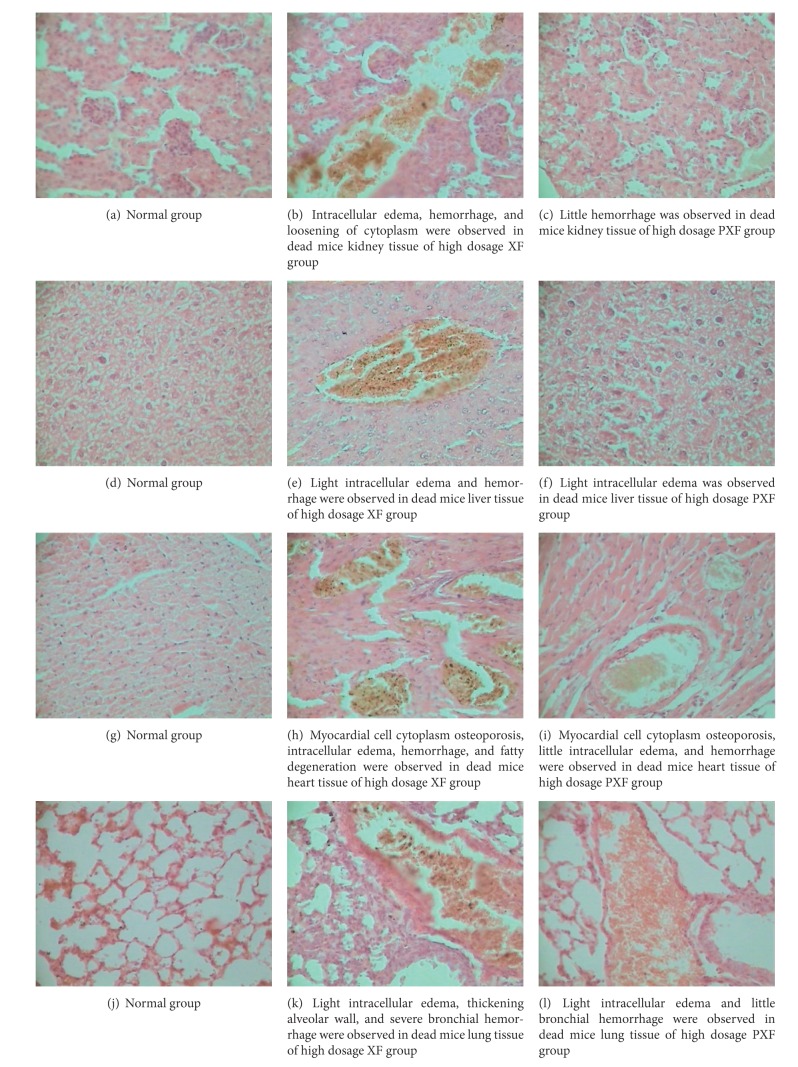
Comparison of microscopic morphology in kidney (a, b, and c), liver (d, e, and f), heart (g, h, and i), and lung (j, k, and l) tissue between control group (a and d), high dosage XF group (b and e), and high dosage PXF group (c and f). Original magnification ×400.

Postmortem examination did not reveal any gross abnormality of the brain and the organs of the chest and abdominal cavities in all treated groups. Liver and kidney pathological examination of dead mice showed the congestion and focal necrosis in the liver and renal tubules (Figure 4). Pathological damage in the heart and lung in high dosage XF and PXF groups was reported for the first time.

Blood samples of high dosage XF and PXF groups were hard to collect due to the close death time. Therefore, blood cell analysis was conducted only in the middle and low dosage XF and PXF groups (Table 5). Most of the blood cell indexes, such as white blood cells (WBC), red blood cells (RBC), haemoglobin (HGB), and platelet (PLT), in low dosage XF and PXF groups remained at normal level compared with control group. However, the PLT content decreased significantly in high dosage PXF group. This indicated that thrombocytopenia might be an obvious adverse effect of XF.

Judging from the mortality rate, toxicity symptoms and signs, tissue morphology, and blood cell analysis results, neither XF nor PXF showed acute toxicity in the low dosage group. That indicated that the single dose administration of XF or PXF was safe even at 10 times of the allowed human highest daily dosage. However, higher dosages of XF and PXF both displayed obvious acute toxicity. 40% of the mice in high dosage PXF group and 80% of the mice in high dosage XF group were dead with severe kidney, liver, heart and lung morphology changes within the 7-day observation period. Fortunately, PXF showed more moderate acute toxicity effects than XF in the same dosage level.

### 3.4. *In Vivo *Chronic Toxicity of XF and PXF

12-week administrations of water extraction of XF and PXF in low, middle, and high dosage were conducted in order to investigate their chronic toxicity. Taking into account the clinic dosage, the low, middle, and high dosages of this chronic toxicity research were set as 0.585, 1.755 and 5.265 g/kg body weight.

Nineteen rats were dead during the research procedure (Table 6). Nine of them were dead immediately after our experimental operations, such as excessive anesthesia and blood sample collection procedure. These deaths might be most probably related to the experimental operations. The other 10 rats were dead probably due to the administrations of XF and PXF. Most of the 10 rats were dead during the 10th to 12th weeks. Four of them were in XF treatment groups and 6 of them were in PXF groups. Four of the 10 rats were female, while 6 of the 10 rats were male. A total of 1, 2, and 1 rats were dead in the low, middle, and high dosage groups, respectively. A total of 4, 1 and 1 rats were dead in the low, middle, and high dosage groups, respectively. No obvious dose-toxicity relationship was observed.

**Figure 5:**
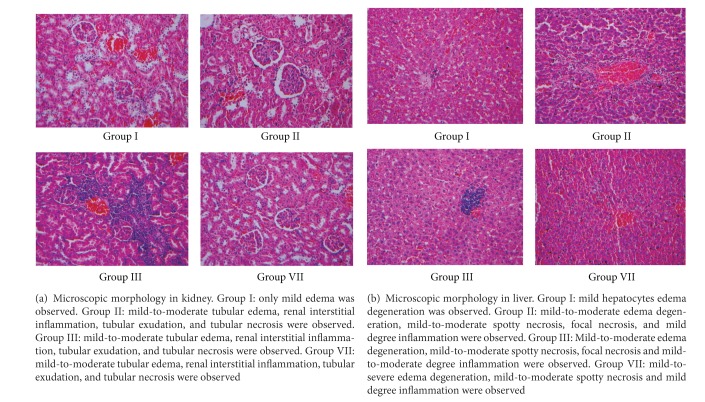
Comparison of microscopic morphology in kidney (a) and liver (b) tissue between Group I (control group), Group II (low dosage of XF), Group III (middle dosage of XF), and Group VII (high dosage of PXF). Original magnification ×200.

Postmortem examination did not reveal any gross abnormality of the brain and the organs of the chest and abdominal cavities. Histopathological examinations of kidney, liver, heart and lung samples were all carried out. However, no obvious pathological changes were found in the heart and lung samples. Liver and kidney pathological examination of dead mice showed congestion and focal necrosis in the liver and renal tubules (Table 7 and Figure 5) without a dose-dependent relationship.

**Figure 6:**
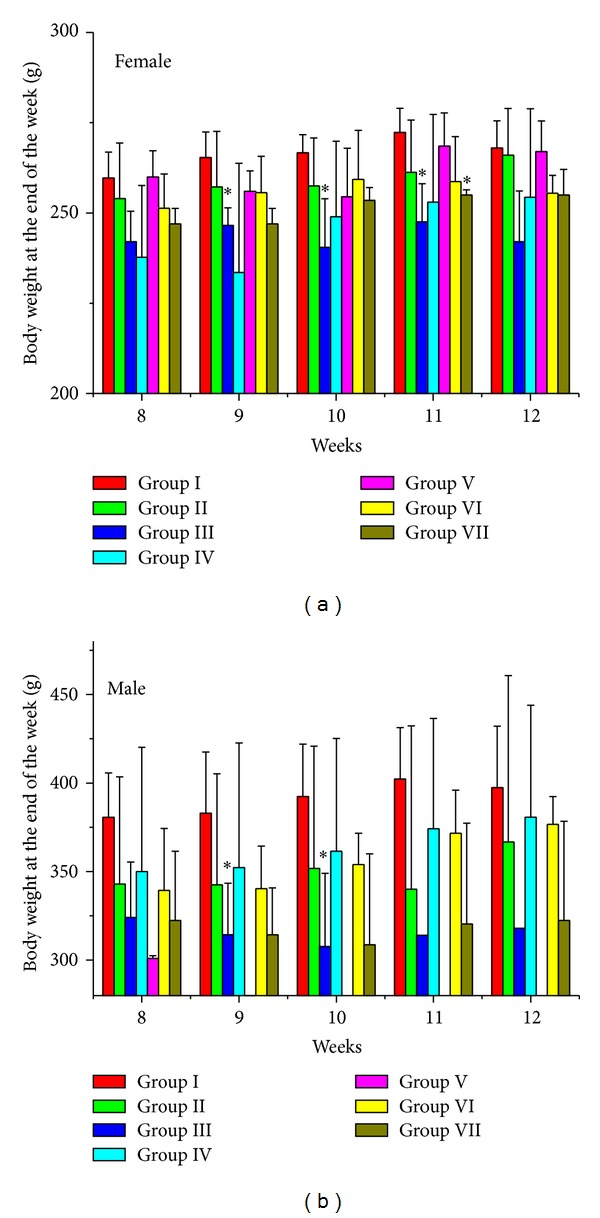
Female and male rats' body weights at the end of the 8th to 12th weeks. The ∗ indicates a significant difference compared with control group, *P < 0.05.

No significant body weight changes were observed in both male and female rats in the first 8 weeks of this research (data not shown). Obvious body weight changes were found after the end of the 8th week (Figure 6). Body weights (BWs) of female rats in all treated groups were reduced, although only female rats in Group III (9th, 10th, and 11th weeks) and Group VII (11th week) were significantly lighter than those in Group I (Control I) in statistical level. Body weight losses were more conspicuous in male rats groups. The mean body weight of Group III was about 80% of the control group.

Blood cell analysis was conducted in all treatment groups from the 3rd to the 12th weeks (data not shown); however, no significant alterations were observed in all groups compared to the control group. This indicated that the 3-month administration would not affect the composition and content of basic blood cell, such as WBC, RBC, HGB, and PLT.

**Figure 7:**
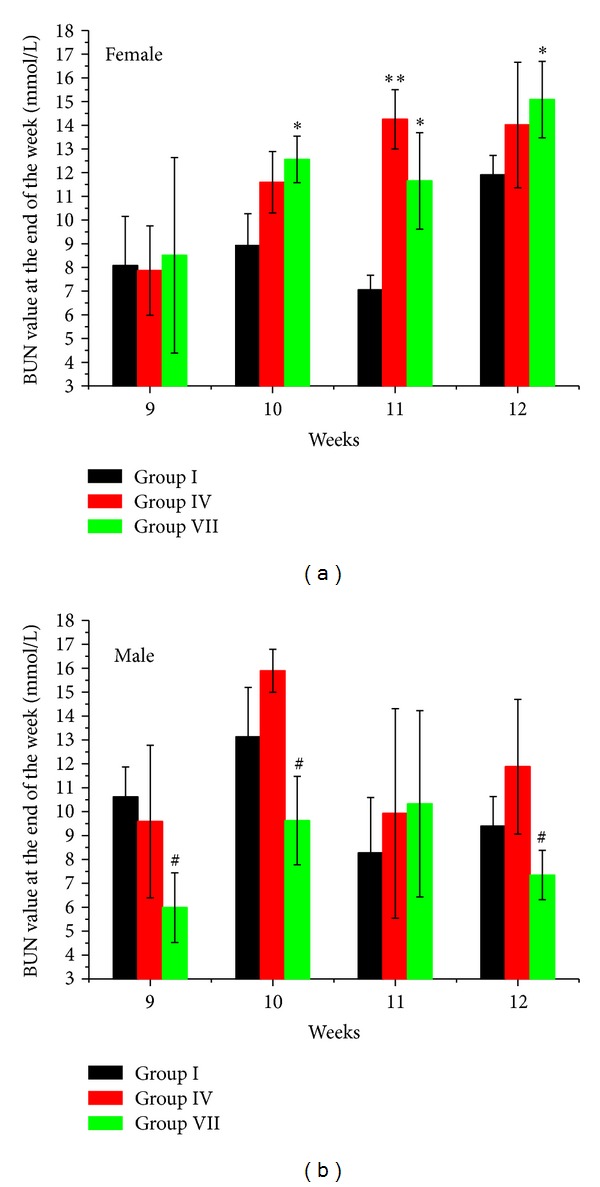
Female and male rats' BUN values at the end of the 9th to 12th weeks. The ∗ indicates a significant difference compared with control group, *P < 0.05, **P < 0.01, and ***P < 0.001. The # indicates a significant difference compared with XF groups in the same dosage, ^#^ *P* < 0.05, ^##^ *P* < 0.01, and ^###^ *P* < 0.001. Only BUN values in high dosage XF and PXF groups were shown in this figure.

**Figure 8:**
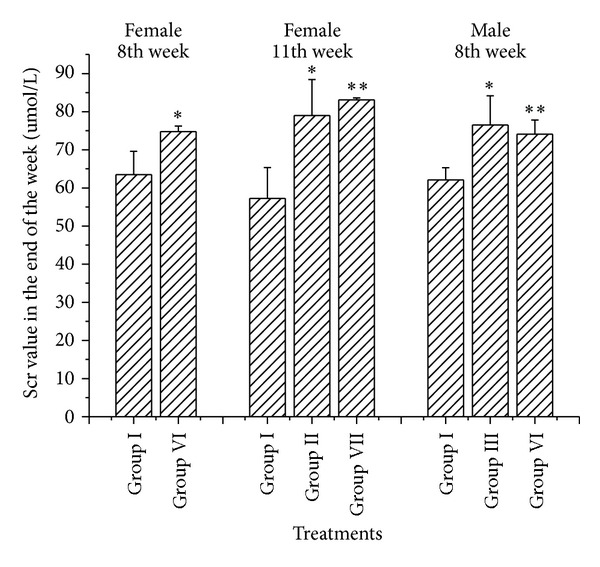
Female and male rats' Scr values at the 8th and 11th weeks. The ∗ indicates a significant difference compared with control group, *P < 0.05, **P < 0.01, and ***P < 0.001. Only Scr values in partly treatment groups with significant alterations were shown in this figure.

Scr and BUN were analyzed from the 3rd to the 12th weeks. Similar change trends were observed in Scr and BUN results (in Figures 7 and 8 and Supplementary Tables 1–4 in Supplementary Material available online at http://dx.doi.org/10.1155/2013/403491). Increasing BUN compared to Group I (control group) was observed only in female rats in Group IV (high dosage XF) and Group VII (high dosage PXF) from the 10th to the 12th weeks (Figure 7). Sporadic and transient Scr increases appeared only in the 8th (Group VI) and the 11th (Groups II and VII) weeks for female rats and the 8th (Groups III and VI) weeks for male rats (Figure 8). BUN values were even decreased in male rats in Group VII.

In conclusion, low and middle dosage of XF and PXF would not induce serum BUN alteration even in a three-month chronic research. High dosage PXF even decreased the BUN compared to high dosage XF in male rats in the 9th, 10th, and 11th weeks. Scr alterations were not sustained but irreversible in both female and male rats in all dosage XF and PXF groups.

As early indicators of renal fibrosis, serum HA, LN, and HPCIII content values were also tested in our research (Tables 8, 9, and 10). Some transient increasing of them were found. Although no HA, LN, and HPCIII increase was found at the end of the research, we still noticed that serum HA and HPCIII values were sustained increasing from the 4th week to the 8th week in Group V male rats. Whether this increase is related to the death of these rats still need to be investigated. That indicated that the renal fibrosis risks still existed although no fibrosis was found in the pathological examination of liver and kidney.

## 4. Discussion

Adverse effects of Xanthii Fructus were frequently reported in recent years, although the toxicity of Xanthii Fructus is clearly alerted in the Pharmacopoeia of China (2010 edition). Moreover, its basic toxicity data and related mechanism were not well investigated. In this manuscript, we systemically evaluated the *in vitro* and *in vivo* toxicity of Xanthii Fructus, its processed product, and its major chemical constituent.

In the HK-2 renal cells, water extraction of XF displayed no cell membrane damage effects even in the highest 100 *μ*g/mL concentration; however, it might affect the function of renal cell mitochondria.

Atractyloside was frequently reported to be a toxic substance of Xanthii Fructus. The toxic mechanisms of atractyloside were also well explained. Atractyloside competitively inhibits the adenine nucleoside carrier in isolated mitochondria and thus blocks oxidative phosphorylation [10]. Atractyloside inhibits gluconeogenesis and fatty acid oxidation but accelerates anaerobic glycolysis and glycogenolysis. The metabolic alterations in humans and animals include an acute hyperglycaemic phase, due to rapid depletion of skeletal muscle and hepatic glycogen, inhibition of glycogen synthesis, and a subsequent marked hypoglycaemic phase. The rapidly declining plasma glucose resulted in insufficient glucose delivery to the brain to sustain normal functions. As the severe hypoglycaemia persisted, depression of respiration, hypoxaemia, tissue hypoxia (diminished oxygen consumption), acidosis, convulsion, coma, and, in some cases, death followed. However, in this experiment, atractyloside did not show such obviously toxic effects as other published researches. We consulted this to the differences between *in vitro* and *in vivo* research results. Limited metabolic enzyme and limited exposure time might restrict the exposure of atractyloside toxicity. Moreover, toxic effects induced by atractyloside might be mediated by its *in vivo* metabolite, which could not happen in our *in vitro *experiment system.

*In vivo* toxicity assays provided more information and basic data for the clinical usage of Xanthii Fructus. Both XF and PXF displayed severe acute toxicity in 240 times of human highest dosage; however, this dosage was too extreme in the clinical usage. Fortunately, the single dose administration of XF and PXF was safe even at 10 times of allowed human highest daily dosage. No obvious behavioral changes, blood cell analysis results changes, and general organ lesions were observed in this dosage.

Chronic toxicity assays focused on the more common dosage with high probability of occurrence in clinical practice. In order to evaluate their toxicity to kidney, BUN and Scr were tested as the function index of kidney. The BUN test, along with the creatinine test, is primarily used to evaluate kidney function in a wide range of circumstances to help diagnose kidney disease and to monitor people with acute or chronic kidney dysfunction or failure. Increased BUN levels suggest impaired kidney function. This may be due to acute or chronic kidney disease, damage, or failure. It may also be due to a condition that results in decreased blood flow to the kidneys, such as congestive heart failure, shock, stress, recent heart attack, or severe burns, due to conditions that cause obstruction of urine flow, or due to dehydration. Moreover, HA, LN, and PCIII, the major ingredients of extracellular matrix (ECM), were also evaluated. Synthesis and accumulation of large quantities ECM were foundations of kidney fibrosis.

Twelve-week administrations of low, middle and high dosage of XF and PXF were performed in SD rats. Notable changes in body weight and blood cell and BUN and Scr changes occurred sporadically in middle and high dosage groups after the 9th week. Although some liver and kidney pathological damage was found in the high dosage groups, no early stage changes of renal fibrosis were found.

All the above results indicated that the single use of even 10 times the permitted highest dosage of XF and PXF is safe. However, long-term usage of overdose XF and PXF was not entirely safe. Adverse effects or even death casesoccurred after continuous administration of 3 times or 9 times the permitted dosage of XF and PXF for 2 months.

## Supplementary Material

Supplement Tables 1-4 provided the data of BUN and Scr values in female and male rats in chronic toxicity assays, respectively.

## Figures and Tables

**Table 1:** Mice grouping and treatments in acute toxicity testing.

Groups	Treatment	Dosage (g/kg body weight)	Relative ratio to human highest daily dosage
A	Physiological saline	0	—
B	Water extraction of XF	16.68	10 times
C	Water extraction of XF	166.8	100 times
D	Water extraction of XF	400	240 times
E	Water extraction of PXF	16.68	10 times
F	Water extraction of PXF	166.8	100 times
G	Water extraction of PXF	400	240 times

**Table 2:** Rats grouping and treatments in chronic toxicity testing.

Groups	Treatment	Dosage (g/kg body weight)	Relative ratio to human median daily dosage
I	Physiological saline	0	—
II	Water extraction of XF	0.585	1 time
III	Water extraction of XF	1.755	3 times
IV	Water extraction of XF	5.265	9 times
V	Water extraction of PXF	0.585	1 time
VI	Water extraction of PXF	1.755	3 times
VII	Water extraction of PXF	5.265	9 times

**Table 3:** MTT survival rate and LDH leakage rate of HK-2 cell exposed to atractyloside potassium salt (AL).

Exposure (*μ*M)	MTT survival rate (%)	LDH leakage rate (%)
Control	100	7.63 ± 0.00
HgCl_2_	5.39 ± 1.48	95.3 ± 19.3
5	88.4 ± 13.2	8.99 ± 0.311
10	93.4 ± 8.68	8.22 ± 0.571
20	89.2 ± 4.42	8.96 ± 0.198
40	92.2 ± 2.82	9.28 ± 0.421
80	99.5 ± 15.33	10.3 ± 1.25
160	94.1 ± 10.3	8.73 ± 0.132
240	98.2 ± 6.56	9.62 ± 0.744
320	92.4 ± 3.12	11.7 ± 0.981

**Table 4:** Mice grouping, treatments, and responses in acute toxicity testing.

Groups	Treatment	Dosage (g/kg)	Male		Female	
			Mortality	Symptoms and signs	Mortality	Symptoms and signs
A	Physiological saline	0	0	None	0	None
B	Water extraction of XF	16.68	0	None	0	None
C	Water extraction of XF	166.8	0	Slow respiratory frequency (20%), slow action (20%)	0	Salivation (20%), piloerection (20%), fast respiratory frequency (20%), slow action (20%)
D	Water extraction of XF	400	80%	Vomiting (20%), writhing reaction (20%), fast respiratory frequency (20%), convulsions (20%)	80%	Pupil dilation (20%), piloerection (20%), convulsions (20%)
E	Water extraction of PXF	16.68	0	None	0	None
F	Water extraction of PXF	166.8	0	Dark hair (20%), salivation (20%)	0	Convulsions (20%), salivation (20%)
G	Water extraction of PXF	400	40%	Convulsions (20%), salivation (20%)	40%	Convulsions (20%)

**Table 5:** Blood cell analysis of middle and low dosage XF and PXF groups in acute toxicity assay.

Groups	Treatment	WBC (10^9^/L)	RBC (10^12^/L)	HGB (g/L)	PLT (10^9^/L)
A	Physiological saline	2.13 ± 2.05	5.68 ± 1.07	126 ± 25.4	423 ± 152
C	Water extraction of XF (L)	2.36 ± 0.93	6.05 ± 0.448	162 ± 88.0	343 ± 115
D	Water extraction of XF (M)	3.18 ± 1.55	6.42 ± 0.697	142 ± 22.3	335 ± 128
F	Water extraction of PXF (L)	3.39 ± 1.32	6.58 ± 0.649^#^	150 ± 20.8	347 ± 130
G	Water extraction of PXF (M)	1.82 ± 1.12^#^	5.17 ± 1.04^#^	113 ± 28.1^#^	215 ± 72.5^∗∗#^

*A significant difference compared with control group, **P < 0.01.

^#^A significant difference compared with XF groups in the same dosage, ^#^ *P* < 0.05.

**Table 6:** Rats mortality in chronic toxicity assays.

Groups	Treatment	Mortality	
		Female	Male
I	Physiological saline	None	None

II	Water extraction of XF	None	1^▲^. One dead at the 12 W + 3 D with 266 g BW (BW reduced gradually).

III	Water extraction of XF	1^△^. One dead immediately after the blood sample collection procedure 2 W + 1 D. 2^△^. Another one dead due to excessive anesthesia at the 8 W + 1 D.	1^▲^. One dead at the 11 W + 3 D with 340 g BW (BW reduced gradually). 2^▲^. One dead at the 11 W + 4 D with 261 g BW (BW reduced gradually). 3^△^. Another one dead immediately after the blood sample collection procedure at the 3 W + 1 D.

IV	Water extraction of XF	1^△^. One dead at the 10 W + 7 D with 170 g BW (BW reduced gradually).	None

V	Water extraction of PXF	1^▲^. One dead at the 10 W + 2 D with 248 g BW (normal BW). 2^△^. Another one dead immediately after the blood sample collection procedure at the 3 W + 1 D.	1^△^. One dead immediately after the blood sample collection procedure at the 10 W + 1 D. 2–4^▲^. The other three rats were dead at the 7 W + 6 D (283 g BW), 9 W + 4 D (281 g BW), and 10 W + 4 D (242 g BW), respectively. Their BWs were reduced gradually.

VI	Water extraction of PXF	1^▲^. One dead at the 12 W + 1 D with 271 g BW (normal BW). 2^△^. Another one dead immediately after the blood sample collection procedure at the 2 W + 1 D.	1^△^. One dead immediately after the blood sample collection procedure at the 2 W + 1 D.

VII	Water extraction of PXF	1^▲^. One dead at the 2 W + 6 D with 152 g BW (BW reduced dramatically). 2^△^. Another one dead due to excessive anesthesia at the 8 W + 1 D.	1^△^. One dead immediately after the blood sample collection procedure at the 6 W + 1 D.

Total		9	11

BW: body weight.

2 W + 1 D: the first day of the second week, and so forth.

^△^Deaths of these rats might be most probably related to the experimental operations.

^▲^Deaths of these rats might be most probably related to the administrations of XF and PXF.

**Table 7:** Kidney and liver pathological damage occurrence rates (%) in chronic toxicity assays.

Groups	I	II	III	IV	V	VI	VII
Kidney							
Tubular edema	0	25	50	0	33	0	50
Renal interstitial Inflammatory infiltration	0	50	75	0	33	0	25
Renal tubular exudation	0	50	75	50	33	0	25
Tubular necrosis	0	25	50	0	33	0	25
Fibrosis	0	0	0	0	0	0	0
Liver							
Liver cell edema	0	25	0	25	50	75	25
Spotty necrosis	0	75	100	0	50	0	25
Focal necrosis	0	25	50	0	25	0	0
Inflammation	0	0	50	0	25	0	0
Fibrosis	0	0	0	0	0	0	0

**Table 8:** Serum HA value at the end of the 4th, 8th, and 12th weeks in chronic toxicity assays (ng/mL).

Weeks	Female			Male		
	4th	8th	12th	4th	8th	12th
I	80.2	53.7	55.3	94.0	56.7	57.3
II	60.7	61.9	60.8	61.5	57.2	60.1
III	106	56.8	58.4	64.8	60.2	61.6
IV	53.0	62.3	52.0	55.9	61.5	53.2
V	54.1	57.9	62.2	227	509	None
VI	208	53.5	61.3	56.9	87.3	53.3
VII	76.2	59.0	58.5	61.2	52.8	53.1

None of the results were obtained in Group V male rats due to the fact that all rats were dead at the end of the 10th week.

**Table 9:** Serum LN value at the end of the 4th, 8th, and 12th weeks in chronic toxicity assays (ng/mL).

Weeks	Female			Male		
	4th	8th	12th	4th	8th	12th
I	352	625	368	500	671	309
II	580	551	333	673	484	327
III	559	434	502	1690	390	411
IV	470	403	459	528	383	388
V	547	935	378	436	721	None
VI	2876	347	69	700	454	284
VII	660	552	479	1093	381	253

None of the results were obtained in Group V male rats due to the fact that all rats were dead at the end of the 10th week.

**Table 10:** Serum HPCIII value at the end of the 4th, 8th, and 12th weeks in chronic toxicity assays (ng/mL).

Weeks	Female			Male		
	4th	8th	12th	4th	8th	12th
I	3.39	0.974	3.16	9.14	2.27	1.15
II	1.38	1.48	3.71	2.48	0.528	ND
III	1.32	0.758	1.44	8.05	2.45	3.96
IV	0.986	3.10	1.10	1.16	10.8	8.46
V	7.05	1.71	3.96	9.43	55.7	None
VI	6.63	8.38	1.74	7.46	46.0	7.21
VII	3.91	1.33	0.757	1.57	6.50	8.68

None of the results were obtained in Group V male rats due to the fact that all rats were dead at the end of the 10th week.

ND: not detected.

## Abbreviations:

AL: Atractyloside potassium salt
BUN: Blood urea nitrogen
BW: Body weight
DMSO: Dimethylsulfoxide
ECM: Extracellular matrix
FBS: Fetal bovine serum
LDH: Lactate dehydrogenase
HA: Hyaluronic acid
HGB: Haemoglobin
HPCIII: Human procollagen type III
LD_50_: Median lethal dose
LN: Laminin
MTT: 3-(4,5)-Dimethylthiahiazo(-z-y1)-3,5-di-phenytetrazoliumromide
NRU: Neutral red uptake assay
PLT: Platelet
PXF: Processed Xanthii Fructus
RBC: Red blood cells
RPMI 1640: Roswell Park Memorial Institute medium 1640
Scr: Serum creatinine
WBC: White blood cells
XF: Xanthii Fructus.
