# Supplementary material for: In Vitro Cytotoxicity and In Vivo Acute and Chronic Toxicity of Xanthii Fructus and Its Processed Product
Source: Biomed Res Int. 2013 Nov 26;2013:403491. doi: 10.1155/2013/403491 (PMC3858965; doi:10.1155/2013/403491)
Supplement: Supplementary file 1 — Supplement Tables 1-4 provided the data of BUN and Scr values in female and male rats in chronic toxicity assays, respectively. [file 403491.f1.doc]

**Supplement**

**Supplement Table 1. BUN value of female rats in chronic toxicity assays（X±SD，n≥2）（mmol/L）**

| Weeks | Ⅰ | Ⅱ | Ⅲ | Ⅳ | Ⅴ | Ⅵ | Ⅶ |
| --- | --- | --- | --- | --- | --- | --- | --- |
| 3 | 8.53±1.16 | 9.78±0.616 | 9.74±1.04 | 9.37±0.674 | 8.21±1.08 | 10.8±2.60 | 9.55±2.09 |
| 4 | 8.76±1.00 | 8.78±0.613 | 8.77±0.0808 | 9.49±0.951 | 7.82±1.31 | 8.14±1.26 | 7.27±0.672 |
| 5 | 10.6±1.11 | 9.40±1.25 | 8.04±1.72 | 9.58±0.913 | 8.78±0.358 | 9.81±1.15 | 7.61±1.15 |
| 6 | 9.16±1.52 | 8.09±1.12 | 7.41±0.555 | 9.48±1.88 | 8.89±1.21 | 8.62±0.530^#^ | 8.62±0.877 |
| 7 | 8.15±1.73 | 8.07±0.579 | 7.31±0.313 | 7.86±0.586 | 6.77±1.11 | 7.19±0.810 | 7.84±0.240 |
| 8 | 7.46±1.41 | 6.19±0.0922 | 5.93±1.23 | 7.00±0.323 | 6.68±1.46 | 7.19±1.65 | 9.03±1.75 |
| 9 | 8.08±2.07 | 9.33±3.49 | 8.28±0.509 | 7.87±1.89 | 7.19±0.750 | 8.33±2.61 | 8.51±4.13 |
| 10 | 8.92±1.35 | 9.48±1.22 | 9.23±1.55 | 11.60±1.29 | 9.79±2.21 | 11.5±3.12 | 12. 6±0.976^*^ |
| 11 | 7.06±0.615 | 8.23±3.19 | 6.57±0.707 | 14.25±1.25^**^ | 5.63±3.62 | 5.34±1.12 | 11.6±2.04^*^ |
| 12 | 11.9±0.820 | 12.2±1.27 | 11.29±0.651 | 14.01±2.65 | 9.54±1.53 | 8.51±1.18 | 15.1±1.61^*^ |

The ^*^ indicates a significant difference compared with control group, ^*^ *P* < 0.05, ^**^ *P* < 0.01, ^***^ *P* < 0.001.

The ^#^ indicates a significant difference compared with XF groups in the same dosage, ^#^ *P* < 0.05, ^##^ *P* < 0.01, ^###^ *P* < 0.001.

**Supplement Table 2. BUN value of male rats in chronic toxicity assays（X±SD，n≥2）（mmol/L）**

| Weeks | Ⅰ | Ⅱ | Ⅲ | Ⅳ | Ⅴ | Ⅵ | Ⅶ |
| --- | --- | --- | --- | --- | --- | --- | --- |
| 3 | 7.75±1.26 | 8.56±0.375 | 8.21±0.711 | 9.42±1.22 | 9.62±0.361 | 8.38±0.654 | 7.97±0.540 |
| 4 | 7.71±1.08 | 8.60±0.555 | 8.07±0.423 | 8.88±1.18 | 7.69±0.695 | 7.04±0.305^#^ | 8.06±0.419 |
| 5 | 7.86±0.714 | 7.80±0.838 | 8.03±1.28 | 7.86±1.39 | 7.47±0.779 | 7.09±0.433 | 7.87±0.494 |
| 6 | 8.23±0.624 | 8.28±0.368 | 8.05±0.467 | 7.94±0.467 | 8.25±1.03 | 8.23±0.210 | 8.33±0.690 |
| 7 | 7.41±0.789 | 7.41±0.668 | 7.01±0.499 | 7.04±0.234 | 5.89±0.116 | 7.68±0.822 | 7.80±0.525 |
| 8 | 5.68±1.67 | 4.60±0.839 | 4.03±0.887 | 5.54±0.378 | 5.28±2.59 | 4.84±1.87 | 4.86±1.03 |
| 9 | 10.6±1.25 | 10.2±2.09 | 7.42±2.64 | 9.59±3.19^#^ | 10.2±2.29 | 8.13±2.40 | 5.99±1.46^#^ |
| 10 | 13.1±2.07 | 12.2±2.06 | 13.2±2.94 | 15. 9±0.899^#^ | None | 11.0±3.62 | 9.63±1.84^#^ |
| 11 | 8.27±2.23 | 10.1±3.52 | 11.5 | 9.92±4.38 | None | 11.5±2.61 | 10.3±3.90 |
| 12 | 9.39±1.24 | 10.3±1.31 | 9.30 | 11.9±2.82^#^ | None | 8.50±2.92 | 7.35±1.03^#^ |

The ^*^ indicates a significant difference compared with control group, ^*^ *P* < 0.05, ^**^ *P* < 0.01, ^***^ *P* < 0.001.

The ^#^ indicates a significant difference compared with XF groups in the same dosage, ^#^ *P* < 0.05, ^##^ *P* < 0.01, ^###^ *P* < 0.001.

None results were obtained in Group V male rats due to all rats were dead at the end of the 10^th^ week.

Only one rat in Group III were survived in the 11^th^ and 12^th^, therefore, no SD could be calculated in this group.

**Supplement Table 3. Scr value of female rats in chronic toxicity assays（X±SD，n≥2）（μmol/L）**

| Weeks | Ⅰ | Ⅱ | Ⅲ | Ⅳ | Ⅴ | Ⅵ | Ⅶ |
| --- | --- | --- | --- | --- | --- | --- | --- |
| 3 | 68.7±0.833 | 68.1±3.97 | 68.2±2.67 | 68.1±5.39 | 64.2±1.46 | 68.8±2.82 | 66.1±4.82 |
| 4 | 69.9±8.03 | 71.0±4.70 | 67.2±0.595 | 70.3±6.05 | 63.9±4.92 | 67.2±4.97 | 63.8±1.29 |
| 5 | 74.7±8.06 | 75.0±1.65 | 69.8±6.51 | 79.9±3.06 | 75.3±7.95 | 75.1±3.08 | 71.9±2.42 |
| 6 | 62.2±2.58 | 63.3±5.35 | 53.8±14.3 | 62.7±4.57 | 61.3±3.19 | 60.0±2.25 | 66.2±0.269 |
| 7 | 72.2±5.05 | 73.8±4.87 | 75.6±2.06 | 78.6±3.67 | 71.1±3.75 | 69.5±6.43 | 73.3±18.7 |
| 8 | 63.5±6.04 | 68.9±4.26 | 65.6±5.13 | 72.4±2.19 | 66.5±4.53 | 74.8±1.45^*#^ | 77.4±0.163 |
| 9 | 69.6±18.6 | 67.1±16.7 | 52.3±5.44 | 74.3±17.7 | 65.6±7.12 | 71.3±26.7 | 67.3±1.36 |
| 10 | 79.8±13.2 | 76.0±11.6 | 87.8±10.7 | 83.8±14.0 | 76.5±2.88 | 68.8±5.54 | 75.5±0.127 |
| 11 | 57.3±8.07 | 79.0±9.43^*^ | 71.0±6.31 | 79.2±22.1 | 65.8±27.9 | 50.8±4.25^#^ | 83.1±0.523^**^ |
| 12 | 52.4±15.5 | 56.8±6.24 | 60. 7±6.82 | 60.4±17.7 | 65.2±12.2 | 71. 7±14.7 | 75.0±5.78 |

The ^*^ indicates a significant difference compared with control group, ^*^ *P* < 0.05, ^**^ *P* < 0.01, ^***^ *P* < 0.001.

The ^#^ indicates a significant difference compared with XF groups in the same dosage, ^#^ *P* < 0.05, ^##^ *P* < 0.01, ^###^ *P* < 0.001.

**Supplement Table 4. Scr value of male rats in chronic toxicity assays（X±SD，n≥2）（μmol/L）**

| Weeks | Ⅰ | Ⅱ | Ⅲ | Ⅳ | Ⅴ | Ⅵ | Ⅶ |
| --- | --- | --- | --- | --- | --- | --- | --- |
| 3 | 56.0±2.25 | 55.5±3.55 | 62.9±5.40 | 61.2±2.19 | 61.8±5.08 | 63.6±4.37^*^ | 63.1±5.58 |
| 4 | 62.5±2.96 | 58.8±7.80 | 58.2±5.96 | 64.9±3.93 | 64.2±1.12 | 65.8±4.36 | 59.6±9.08 |
| 5 | 67.2±2.97 | 67.7±5.82 | 65.6±0.613 | 69.1±7.78 | 63.8±4.37 | 68.5±5.91 | 66.5±7.89 |
| 6 | 61.4±5.63 | 62.0±10.5 | 61.0±4.70 | 72.4±5.39 | 70.9±5.46 | 68.5±2.52 | 70.2±4.98 |
| 7 | 67.4±6.48 | 62.1±7.58 | 59.2±3.21 | 70.4±5.13 | 73.5±3.55 | 71. 9±3.25^##^ | 55.8±8.52 |
| 8 | 62.1±3.19 | 69.3±7.58 | 76.5±7.68^*^ | 57.0±25.5 | 63.7±4.55 | 74.1±3.65^**^ | 73.5±2.16 |
| 9 | 64.0±24.5 | 61.9±15.7 | 52.6±9.61 | 63.3±19.3 | 65.2±21.5 | 50.0±9.18 | 59.9±8.46 |
| 10 | 82.5±10.8 | 60.7±9.32 | 66.1±15.6 | 71.1±8.72 | None | 66.2±15.2 | 68.3±4.72 |
| 11 | 70.4±14.5 | 69.9±16.9 | 73.5 | 75.3±16.9 | None | 63.18±14.4 | 65.9±6.44 |
| 12 | 68.9±15.6 | 65.2±25.4 | 78.1 | 61.4±18.1 | None | 65.7±2.86 | 57.1±6.89 |

The ^*^ indicates a significant difference compared with control group, ^*^ *P* < 0.05, ^**^ *P* < 0.01, ^***^ *P* < 0.001.

The ^#^ indicates a significant difference compared with XF groups in the same dosage, ^#^ *P* < 0.05, ^##^ *P* < 0.01, ^###^ *P* < 0.001.

None results were obtained in Group V male rats due to all rats were dead at the end of the 10^th^ week.

Only one rat in Group III were survived in the 11^th^ and 12^th^, therefore, no SD could be calculated in this group
